# Synergistic anti-oxidant and anti-inflammatory effects of ceria/resatorvid co-decorated nanoparticles for acute lung injury therapy

**DOI:** 10.1186/s12951-023-02237-y

**Published:** 2023-12-21

**Authors:** Yue Wu, Yawen Zhang, Xuanyu Tang, Shuhui Ye, Jingjing Shao, Linglan Tu, Junzhi Pan, Lingfeng Chen, Guang Liang, Lina Yin

**Affiliations:** 1 Zhejiang TCM Key Laboratory of Pharmacology and Translational Research of Natural Products, School of Pharmacy, Hangzhou Medical College, Hangzhou, 310014 Zhejiang China; 2 Affiliated Yongkang First People’s Hospital and School of Pharmacy, Hangzhou Medical College, Hangzhou, 310013 Zhejiang China; 3 School of Pharmacy, Wenzhou Medical University, Wenzhou, 325035 China

**Keywords:** Acute lung injury, Nano-delivery system, ROS-responsive, Ceria nanoparticles, Resatorvid, Anti-oxidation, Anti-inflammation

## Abstract

**Background:**

Acute lung injury (ALI) is a critical inflammatory response syndrome that rapidly develops into acute respiratory distress syndrome (ARDS). Currently, no effective therapeutic modalities are available for patients with ALI/ARDS. According to recent studies, inhibiting both the release of pro-inflammatory cytokines and the formation of reactive oxygen species (ROS) as early as possible may be a promising therapy for ALI.

**Results:**

In this study, a ROS-responsive nano-delivery system based on oxidation-sensitive chitosan (Ox-CS) was fabricated for the simultaneous delivery of Ce NPs and RT. The in vitro experiments have shown that the Ox-CS/Ceria-Resatorvid nanoparticles (Ox-CS/CeRT NPs) were rapidly and efficiently internalised by inflammatory endothelial cells. Biological evaluations validated the significant attenuation of ROS-induced oxidative stress and cell apoptosis by Ox-CS/CeRT NPs, while maintaining mitochondrial function. Additionally, Ox-CS/CeRT NPs effectively inhibited the release of pro-inflammatory factors. After intraperitoneal (*i.p.*) administration, Ox-CS/CeRT NPs passively targeted the lungs of LPS-induced inflamed mice and released the drug activated by the high ROS levels in inflammatory tissues. Finally, Ox-CS/CeRT NPs significantly alleviated LPS-induced lung injury through inhibiting both oxidative stress and pro-inflammatory cytokine expression.

**Conclusions:**

The created Ox-CS/CeRT NPs could act as a prospective nano-delivery system for a combination of anti-inflammatory and anti-oxidant therapy of ALI.

**Graphical Abstract:**

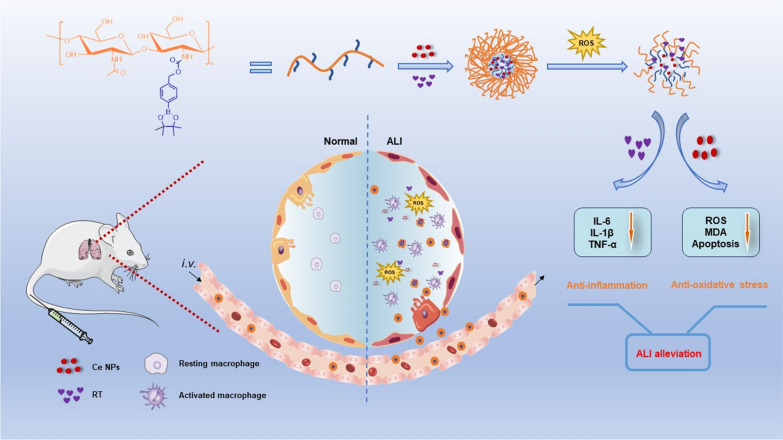

**Supplementary Information:**

The online version contains supplementary material available at 10.1186/s12951-023-02237-y.

## Background

ALI is a pulmonary inflammatory syndrome that is directly or indirectly caused by acute inflammation or other injuries, which may further develop into ARDS and quickly progress to multi-organ failure [1]. Despite the development of advanced technologies, an effective treatment for ALI remains elusive. The mortality of ALI/ARDS is still high [2, 3].

Most research has shown that inflammation plays an essential role in the pathogenesis of ALI [4]. During the progression of ALI, pulmonary macrophages are activated, which recruit neutrophils and circulating macrophages to release numerous proinflammatory factors. Therefore, anti-inflammatory treatment represents a reasonable strategy for ALI. In this aspect, resatorvid (RT) is a selective inhibitor of Toll-like receptor 4 (TLR4) that inhibits pro-inflammatory cytokines production such as IL-6 and TNF-α. Owing to its excellent anti-inflammatory properties, RT has been widely applied to treat various inflammatory diseases [5–7]. Although these preclinical experiments have substantiated anti-inflammatory effects, clinical trials did not yield positive results. This may be due to non-specific distribution, rapid elimination as well as short retention time in lung tissues. Moreover, inflammation is a complex and changeable pathophysiological process. As a result, it appears difficult for a single treatment to provide a satisfactory therapeutic effect. Consequently, combination therapy via different approaches may give a new and effective strategy for the treatment of ALI.

In addition to inflammation disorder, ALI is also closely related to oxidative stress, and the excessive presence of ROS accelerates the development of ALI [4]. Therefore, it is crucial to develop formulations that can effectively inhibit both pro-inflammatory cytokines and ROS for the treatment of ALI. Practically, anti-oxidative drugs like vitamin E and curcumin have been used as ROS scavengers. However, these conventional antioxidants have problems of low target specificity and large antioxidant depletion capability, quite limiting their applications. Recently, nanoenzymes with inherent ROS-scavenging activities have emerged as novel antioxidants to overcome previous failures [8, 9]. Among these, ceria nanoparticles (Ce NPs) have been researched as potent ROS scavengers for a variety of ROS-related disorders, such as sepsis, ischaemic stroke, and Alzheimer’s disease [10, 11]. As a regenerative redox machinery, Ce NPs can exert powerful antioxidative activities. The antioxidative properties of Ce NPs are due to the existence of two reversible oxidation states (Ce^3+^ and Ce^4+^) [12, 13]. Unfortunately, the ultrasmall size of Ce NPs leads to an excessively short half-life in circulation and easy inter-particle aggregation, which can hinder clinical translation. Currently, there are no studies in the literature addressing the use of Ce NPs in combination with drugs for effective combination therapy of ALI.

Nanocarrier has unique benefits in addressing the shortcomings of traditional therapy of ALI by improving short half-life, low delivery efficiency and the water solubility of drugs. In addition, nanoparticles can directly penetrate the dysfunctional endothelium and target lung tissues. Recently, ROS-responsive nanoparticles (ROS-NPs) were developed to respond to physiological oxidative microenvironments [14, 15]. Furthermore, ROS-responsive NPs can be employed to specifically deliver therapeutic drugs to inflamed lung tissues and achieve the on-demand release of drugs according to the ALI microenvironment.

**Scheme 1:**
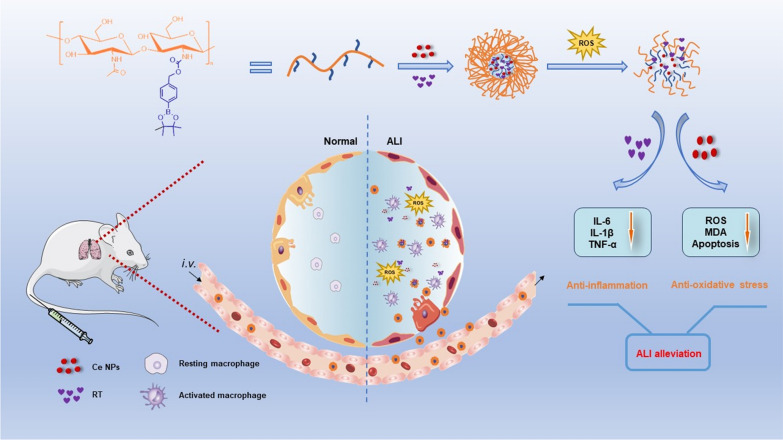
Schematic illustration of Ox-CS/CeRT NPs for acute lung injury

In this study, we synthesized a ROS-responsive material from chitosan (CS), a broadly used compound with excellent biocompatibility and low immunogenicity. As oxidation-responsive units, boronic esters have been recently used as cleavable groups for the preparation of prodrugs, ROS-sensitive imaging probes, and degradable materials [16–18]. As a proof of concept, here 4-phenylboronic acid pinacol ester (PBAP) was chemically conjugated onto hydroxyl groups of chitosan to developed oxidation-sensitive amphiphilic chitosan (Ox-CS) for simultaneous delivery of Ce NPs and RT. As shown in Scheme 1, NPs could be targeted passively to the inflamed lung tissues and rapidly released Ce NPs and RT triggered by ROS in lung tissues. According to our knowledge, Ox-CS/CeRT NPs was supposed to improve the treatment of ALI through combined anti-inflammation and anti-oxidation.

## Materials and methods

### Synthesis and characterization of Ox-CS

Based on a previously reported method [16, 19], 10 mmol of 4-(hydroxymethyl) phenylboronic acid pinacol ester (PBAP) and 50 mmol of *N*,*N*′-carbonyldiimidazole (CDI) were dissolved in dichloromethane (DCM; 20 mL). After reaction for 12 h under nitrogen, CDI-activated PBAP was obtained as the crude product. The samples were washed three times with 20 mL of water, and rinsed with 10 mL of brine, followed by drying over MgSO_4_. Thereafter, 10 mmol of 4-dimethylaminopyridine (DMAP) and 10 mmol of CDI-activated PBAP were added after 5 mmol of chitosan (CS, 89.1% degree of deacetylation, 50 KDa molecular weight) had been mixed in 10 mL of anhydrous dimethyl sulfoxide (DMSO). The mixture was stirred at 25 °C overnight and the final product was obtained by precipitating the mixture in deionized water. The samples were centrifuged, and lyophilised to get pure powders. Successful conjugation was confirmed by nuclear magnetic resonance hydrogen spectroscopy (^1^H-NMR) spectroscopy.

### Synthesis and characterization of Ce NPs

Ce NPs were prepared according to the previously reported hydrolytic sol–gel method [20]. Firstly, cerium (III) acetate sesquihydrate ((Ce (CH_3_CO_2_)_3_·xH_2_O, 99%; 1 mmol) and oleylamine (C18: 80–90%; 12 mmol) were dissolved in xylene (15 mL). Subsequently, the reaction solution was stirred vigorously overnight at 25 °C and then heated to 90 °C at a rate of 2 °C/min under vacuum. To trigger the sol–gel reaction, 1 mL of deionised water was quickly injected into the heated solution. The mixture was then incubated for 3 h until the reaction was terminated with a light-yellow colloid. The reaction solution was cooled to 25 °C and precipitated by adding acetone. Ce NPs were harvested by centrifugation and resuspended in chloroform for subsequent experiments.

The particle size and ζ-potential measurements of Ce NPs were performed on a Zetasizer Nano ZS90 (Malvern, UK). Morphological features were observed by transmission electron microscopy (TEM; HT7700; Hitachi, Japan). The concentration was determined using inductively coupled plasma optical emission spectrometry (ICP-OES; Agilent 720ES, USA). X-ray diffraction patterns (XRD) of samples were obtained using a powder X-ray diffractometer (D8ADVANC; Bruker, Germany). X-ray photoelectron spectroscopy (XPS) for elemental analysis was measured using a VG ESCALAB 220iXL instrument (Thermo Fisher Scientific, USA).

The main ROS, O_2_^**.**^−, and H_2_O_2_, were used to evaluate the ROS scavenging capability of Ce NPs. All experiments were conducted according to the protocols of different assays. The superoxide anion (O_2_^**.**^−) scavenging activity was conducted with a total SOD activity assay kit (Cat. #S0101S; Beyotime Biotechnology, China). The hydrogen peroxide (H_2_O_2_) quenching activity was performed with a catalase assay kit (Cat. #S0051; Beyotime Biotechnology, China).

### Preparation and characterization of Ox-CS/CeRT NPs

Ox-CS/Ce NPs and Ox-CS/CeRT NPs were prepared by the thin film dispersion method. Briefly, 10 mg of Ox-CS and 1 mg of RT were dissolved in 10 mL of methyl alcohol. Next 1 mg of Ce NPs was added and stirred at 25 °C for 10 min. The organic solvent was removed under vacuum in a rotating evaporator for 1 h at 60 °C. The film was hydrated with pH 7.4 buffer at 60 °C for 1 h, followed by ultrasonic treatment for 10 min. Subsequently, the solution was subject to centrifugation. Finally, Ox-CS/Ce NPs or Ox-CS/CeRT NPs were obtained after freeze–drying. NPs loaded with different fluorescent dyes (coumarin-6 and DiR) were prepared using similar procedures.

The particle size distribution, ζ-potential, and polydispersity index (PDI) of the synthesised NPs were measured using a Zetasizer Nano ZS90 (Malvern, UK). The morphology of Ox-CS/Ce NPs and Ox-CS/CeRT NPs was confirmed using transmission electron microscopy (HT7700; Hitachi, Japan).

Fourier transform infrared spectroscopy (FI-IR, Thermo Scientific Nicolet iS20) was used for prove the successful loading of RT. The FT-IR spectra were recorded in transmittance mode with a resolution of 4 cm^−1^ in a scanning range of 3800–500 cm^−1^ for 30 scans at room temperature.

The physical stability of Ox-CS/Ce NPs and Ox-CS/CeRT NPs was examined by monitoring the size changes at different time points under 4 °C storage conditions. The stability of Ox-CS/Ce NPs and Ox-CS/CeRT NPs in serum was also investigated. Freshly prepared Ox-CS/Ce NPs and Ox-CS/CeRT NPs was dispersed in 10 mL pH 7.4 PBS (containing 10% fetal bovine serum) and incubated at 37 °C. Samples were collected at a predetermined time to measure the particle size.

### Drug encapsulation efficiency (EE) and drug loading (DL)

Ox-CS/CeRT NPs (0.5 mL) were diluted to 5 mL with DMSO and sonicated for 10 min to destroy the nanoparticle and release the drug. The obtained suspension was centrifuged and the drug in supernatant was detected via high-performance liquid chromatography (HPLC) at 254 nm. [Ce] concentrations of samples were analyzed using ICP-OES (Agilent 720ES, USA). The HPLC system comprised an SPD-20 A UV–vis detector (Shimadzu, Japan), a binary LC-20AD pump (Shimadzu, Japan), and a Diamonsil™ C_18_ column (5 μm, 250 mm × 4.6 mm). The mobile phase consisted of methanol and 1% trifluoroacetic acid (TFA) (70/30, v/v), and the flow rate was 1 mL/min. The EE % and DL % were calculated using Eqs. (1) and (2), as follows:

1 $${EE\; (\%)=}\frac{{amount\; of\; drug\; in\; NPs}}{{total\; drug\; added}}\times{100 \%},$$

2 $$DL\;(\%)=\frac{{amount\; of\; drug\; in\; NPs}}{{weight\; of\; NPs}}\times{100\%}$$

### In vitro drug release

The release profiles of RT from Ox-CS/CeRT NPs were investigated under different concentrations of H_2_O_2_ (0, 200 and 500 µM, respectively). Briefly, the Ox-CS/CeRT NPs dispersed in deionized water (1 mL; 1 mg/mL) was put in a dialysis bag (MWCO:1 kDa; Wuhan Xinsirui Technology Co., Ltd., China), and subsequently placed in 20 mL of phosphate-buffered saline (PBS, pH 7.4, 1% SDS) with different H_2_O_2_ concentrations. The system was shaken at a rate of 100 rpm while being incubated at 37 °C. At various time intervals, 1 mL of the medium was withdrawn and fresh medium was added. The cumulative drug release rate of RT was measured using HPLC as described above.

### Cell culture and cytotoxicity studies

Human umbilical vein endothelial cells (HUVECs) were obtained from the American Type Culture Collection (ATCC, USA). The cells were cultured in an Endothelial Cell Medium (ECM; Cat. #1001) supplemented with 5% (v/v) foetal bovine serum (FBS; Cat. #0025), 1% Endothelial Cell Growth Supplement (ECGS; Cat. #1052), and 1% Penicillin/Streptomycin Solution (P/S; Cat. #0503). The above cell culture media and additives were purchased from ScienCell (Carlsbad, CA, USA). The cells were incubated at 37 °C in an incubator (5% CO_2_, 90% relative humidity).

CCK-8 assay was utilized with HUVECs to evaluate the cytotoxicity of RT, Ox-CS, Ox-CS/Ce NPs and Ox-CS/CeRT NPs. HUVECs were seeded in 96-well plates (5000 cells/well) and culture for 24 h. Next, the cells were treated with H_2_O_2_ or gradient concentration of drugs (RT, Ox-CS/Ce NPs or Ox-CS/CeRT NPs). After incubating for 24 h, Cell Counting Kit-8 (CCK-8; Biosharp Life Science, China) was used for measuring cell viability.

### Cellular uptake

Coumarin-6 (C6) was used as a fluorescent probe for the cellular uptake studies. HUVECs were seeded in 12-well plates (1 × 10^5^ cells/well) and cultured for 12 h. Subsequently, NPs labelled with coumarin-6 (Ox-CS/C6 NPs) were added and incubated for different times (1, 3, 6 h) at 37 °C. After thoroughly washed with PBS to remove non-uptake NPs, the cells were collected for quantitative flow cytometric analysis (Accuri C6 Plus; BD Biosciences, USA).

For microscopic analysis, the cells were fixed in 4% paraformaldehyde for 15 min after being washed three times with PBS. The nuclei and membranes were stained with DAPI and DiI, respectively. Finally, the cellular fluorescence was observed under a fluorescence microscope (EVOS M7000; Invitrogen, USA).

### In vitro anti-oxidant and anti-inflammatory activities of Ox-CS/CeRT NPs

#### Intracellular ROS measurement

HUVECs were seeded in 12-well plates (1 × 10^5^ cells/well) and cultured for 12 h. The cells were pretreated with different interventions (RT, Ox-CS/Ce NPs, and Ox-CS/CeRT NPs) for 1 h and then incubated with H_2_O_2_ (200 µM) for 12 h. Subsequently, cells were washed three times and treated with 10 µM 2′,7′-dichlorofluorescein-diacetate (DCFH-DA; Cat. #D6470; Solarbio Science & Technology, China) in the dark at 37 °C for 30 min. Finally, fluorescence microscopy was used for observation. The mean fluorescence intensity (MFI) was semi-quantified using ImageJ software (NIH, USA).

#### Anti-inflammatory assay

HUVECs were cultivated for 12 h until adhesion after being seeded in 12-well plates (1 × 10^5^ cells/well). The cells were preincubated with different interventions (RT, Ox-CS/Ce NPs, and Ox-CS/CeRT NPs) for 1 h and then treated with LPS (1 µg/mL) for 4 h. After washing once with PBS, cellular RNA was extracted and analysed for mRNA expression levels of pro-inflammatory cytokines.

### In vitro anti-apoptotic activity of Ox-CS/CeRT NPs

#### Cell apoptosis detected by flow cytometry

The Annexin V-APC/PI apoptosis detection kit (Cat. #AT107; Multi Sciences, China) was used to determine the effect of Ox-CS/CeRT NPs on apoptosis. Specifically, HUVECs were seeded in 12-well plates and incubated overnight. Then, HUVECs were pretreated with different interventions (RT, Ox-CS/Ce NPs, and Ox-CS/CeRT NPs). After incubation for 1 h, cells were treated with H_2_O_2_ (200 µM) for 24 h. After washing with pre-chilled PBS, the cells were resuspended in the binding buffer. Next, 5 µL of Annexin V-APC and 10 µL of PI were added, gently vortexed, and incubated for 5 min in the dark. Finally, the cells’ apoptosis was analysed using flow cytometry, and the experimental data were analysed using FlowJo V10 software (TreeStar Inc., USA).

#### Mitochondrial membrane potential (Δψm) measurement

The JC-1 kit (Cat. #C2006; Beyotime Biotechnology, China) was used to determine the Δψm. HUVECs were pretreated with different interventions (RT, Ox-CS/Ce NPs, and Ox-CS/CeRT NPs) for 1 h and then incubated with H_2_O_2_ (200 µM) for 24 h. The cells were incubated with JC-1 staining working solution at 37 °C for 30 min and observed by a fluorescence microscope. Semi-quantitative analysis was also performed.

### ALI mouse models

Male C57BL/6 mice (weight, 20 ± 2 g) were supplied by the Animal Center of Hangzhou Medical College (Hangzhou, China). The mice were housed in a climate-controlled environment with a 12/12 h light/dark cycle and provided standard food and water.

The LPS-induced ALI mouse model was established as described by our laboratory. Briefly, the anaesthetised mice were fixed, freed from the trachea, and then LPS (5 mg/kg; 055-B5; Sigma, USA) was injected via tracheal drip with a microfeeding needle. The ALI model was established by 6 h of LPS stimulation.

### In vivo biodistribution

To study the in vivo targeting properties of NPs, DiR was used as a fluorescent probe for the in vivo biodistribution studies in healthy and ALI mouse models. ALI mouse model was established as mentioned above. After 6 h, the mice were grouped randomly and *i.v.* injected with DiR or Ox-CS/DiR NPs. At the predetermined time intervals, lung and other major organs (n = 3) were harvested for ex vivo imaging using an IVIS imaging system (PerkinElmer, USA). The fluorescence signal was analysed semi-quantitatively using Living Image™ software (Caliper Life Science, USA).

### In vivo therapeutic efficacy study

The therapeutic effect of Ox-CS/CeRT NPs in vivo was investigated by an LPS-induced ALI mouse model. The mice were randomly divided into five groups (n = 5): (1) control group (CON); (2) LPS group (ALI); (3) LPS + RT group (3 mg/kg); (4) LPS + Ox-CS/Ce NPs group; (5) LPS + Ox-CS/CeRT NPs group (equal to 3 mg/kg RT) by *i.p.* injection, 30 min before the LPS challenge. After 6 h, the mice were anesthetized and the trachea was cannulated. Bronchoalveolar lavage fluid (BALF), serum, and lung tissues were obtained for further research.

### Histology and immunohistochemistry

A portion of lung tissues and other major organs from each experimental group were collected and fixed in 4% paraformaldehyde. The dehydrated and transparent tissues were embedded in paraffin and cut into 5 μm thick sections. Tissue sections were then dried, deparaffinised, and stained with hematoxylin and eosin (H&E).

For immunostaining, tissue sections were deparaffinized and antigenically repaired with 3% H_2_O_2_, sodium citrate buffer. After washing with PBS, all sections were then blocked by 5% bovine serum albumin and incubated with anti-F4/80 primary antibody at 4 °C overnight. Next, the sections were incubated with HRP-labelled secondary antibody for 1 h. All tissue sections were counterstained with hematoxylin and examined under the bright field.

### Measurement of pro-inflammatory factors

The levels of pro-inflammatory factors such as interleukin-6 (IL-6) and tumour necrosis factor-α (TNF-α) in the serum or BALF were measured using ELISA kits (Cat. #88-7324 and #88-7064; Invitrogen, USA) according to manufacturer’s instructions.

### Superoxide dismutase (SOD) and malondialdehyde (MDA) assay

SOD and MDA are the most commonly utilized parameters for evaluating the oxidative stress capacities. The determination of SOD and MDA in the lung tissue was performed according to the experimental instructions (Cat. #A001-3-2 and #A003-1-2; Nanjing Jiancheng Bioengineering Institute, China).

### Evaluation of biosafety

The biosafety of different treatments was also evaluated. After administration of Ox-CS/CeRT NPs in ALI mice, animals were euthanized and blood samples were collected for hematological analysis and quantification of biochemical markers relevant to liver/kidney functions. Major organs including heart, liver, spleen, lung, and kidney were isolated. Histopathological sections were prepared and stained with H&E.

Besides, blood compatibility was also assessed. Isolated rat red blood cells were diluted with normal saline to 2% (v/v) and then mixed with an equal volume of Ox-CS/CeRT NPs normal saline solution. In addition, normal saline and pure water mixed with an equal volume of 2% red blood cell suspension were correspondingly set as the negative and positive control groups, respectively. The samples were incubated for 2 h at 37 °C and centrifuged at 12,000×*g* for 10 min. The supernatant was measured at a wavelength of 541 nm with a microplate reader. The haemolysis ratio was calculated according to Eq. (3):

3 $$\text{Haemolysis}\;\text{ratio} =\frac{\text{A}\;\text{sample}-\text{A}\;\text{negative}\;\text{control}}{\text{A}\;\text{positive}\;\text{control}-\text{A}\;\text{negative}\;\text{control}}\times{100 \%},$$

where A_sample_, A_negative control_, and A_positive control_ refer to the absorbances of the sample, the negative control group, and the positive control group, respectively.

### RNA extraction and quantitative real-time PCR (qPCR)

The gene expression of pro-inflammatory factors in cells and lung tissues was identified by qPCR. The total mRNA in cells and lung tissues was extracted and purified by SteadyPure RNA Extraction Kit (Accurate Biology, China). A NanoDrop 2000 spectrophotometre (Thermo Fisher Scientific, USA) was used for the determination of RNA concentration ng. The target genes expressions (IL-6, TNF-α, and IL-1β) were measured by SYBR-based quantitative PCR analysis (Hieff® qPCR SYBR® Green Master Mix, Yeasen Biotech Co., Ltd, China) and CFX Connect Real-Time System (Bio-Rad, USA). The primer sequences used in this study are listed in Additional file 1: Table S1.

### Statistical analysis

All data are presented as mean ± standard deviation (SD). Statistical analysis was performed using one-way ANOVA, and the results were considered statistically significant when the p-value was < 0.05.

## Results and discussion

### Characterization of Ox-CS

The ROS-sensitive material Ox-CS was synthesized by conjugating a ROS-sensitive unit PBAP onto CS. The synthesis of Ox-CS is shown in Additional file 2: Fig. S1. Structural confirmation of Ox-CS was obtained using ^1^H NMR spectroscopy (Additional file 3: Fig. S2). In the ^1^H-NMR spectra of Ox-CS, the characteristic signals of Ar-H from PBAP at 7.49 and 7.70 ppm could be clearly observed, while those from imidazole at 7.09, 7.62 and 7.63 ppm disappeared. These results indicate the successful formation of Ox-CS.

In an aqueous solution, PBAP aggregates to form a hydrophobic nucleus, which can encapsulate hydrophobic Ce NPs; however, the hydrophilic skeleton of CS diffuses outside. After a simple self-assembly process, NPs with micellar structures were formed.

### Synthesis and characterization of Ce NPs

**Fig. 1:**
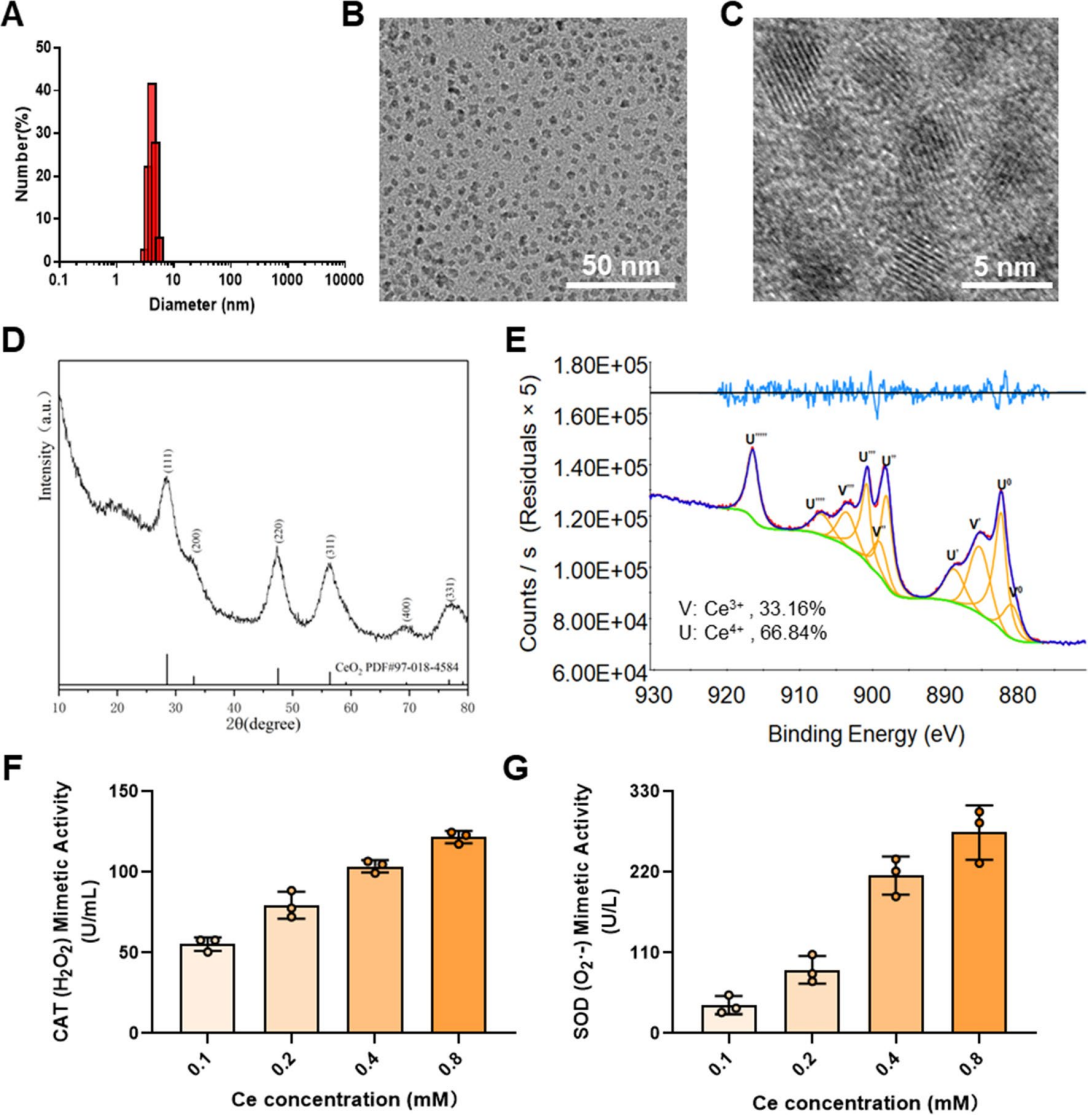
Synthesis and characterization of Ce NPs. **A** Size distribution of Ce NPs. **B**, **C** TEM images, scale bar = 50 nm and 5 nm respectively. **D** XRD analysis and **E** XPS analysis of Ce NPs. ROS scavenging activity of Ce NPs mimics **F** catalase (CAT), and **G** SOD

Ce NPs were synthesized by a sol–gel reaction method. The hydrodynamic particle size of Ce NPs was 3.9 ± 0.5 nm (Fig. 1A). Similarly, TEM imaging revealed ~ 4 nm-sized NPs with clear crystal structure and homogeneous morphology (Fig. 1B, C). The XRD patterns indicated strong diffraction peaks with good peak shapes, suggesting good crystallinity (Fig. 1D). XPS analysis showed that both Ce^3+^ and Ce^4+^ were present on the surface of the Ce NPs. According to the area under the peak of each valence state, Ce^3+^ accounted for 33.16% and Ce^4+^ accounted for 66.84%, respectively (Fig. 1E). The Ce^3+^ site can scavenge ·OH and O_2_^**.**^− through redox reactions or mimic SOD in organisms, whereas the Ce^4+^ site can catalyse the decomposition of H_2_O_2_ by mimicking catalase (CAT) [16]. Such anti-oxidant activity of Ce NPs was measured and all assays confirmed the highly sensitive and concentration-dependent scavenging of ROS by Ce NPs (Fig. 1F, G). A high Ce^3+^ composition has the ability to effectively scavenge a significant amount of ROS. Thus, the ratio of Ce^3+^/Ce^4+^ is important for different ROS-associated diseases. Special attention should be paid to the ratio of Ce^3+^/Ce^4+^ in Ce NPs and their reproducibility. Importantly, the two valence states are interconvertible and cyclically regenerated, providing a chemical basis for catalytic activities [21–23].

### Synthesis and characterization of Ox-CS/CeRT NPs

**Fig. 2:**
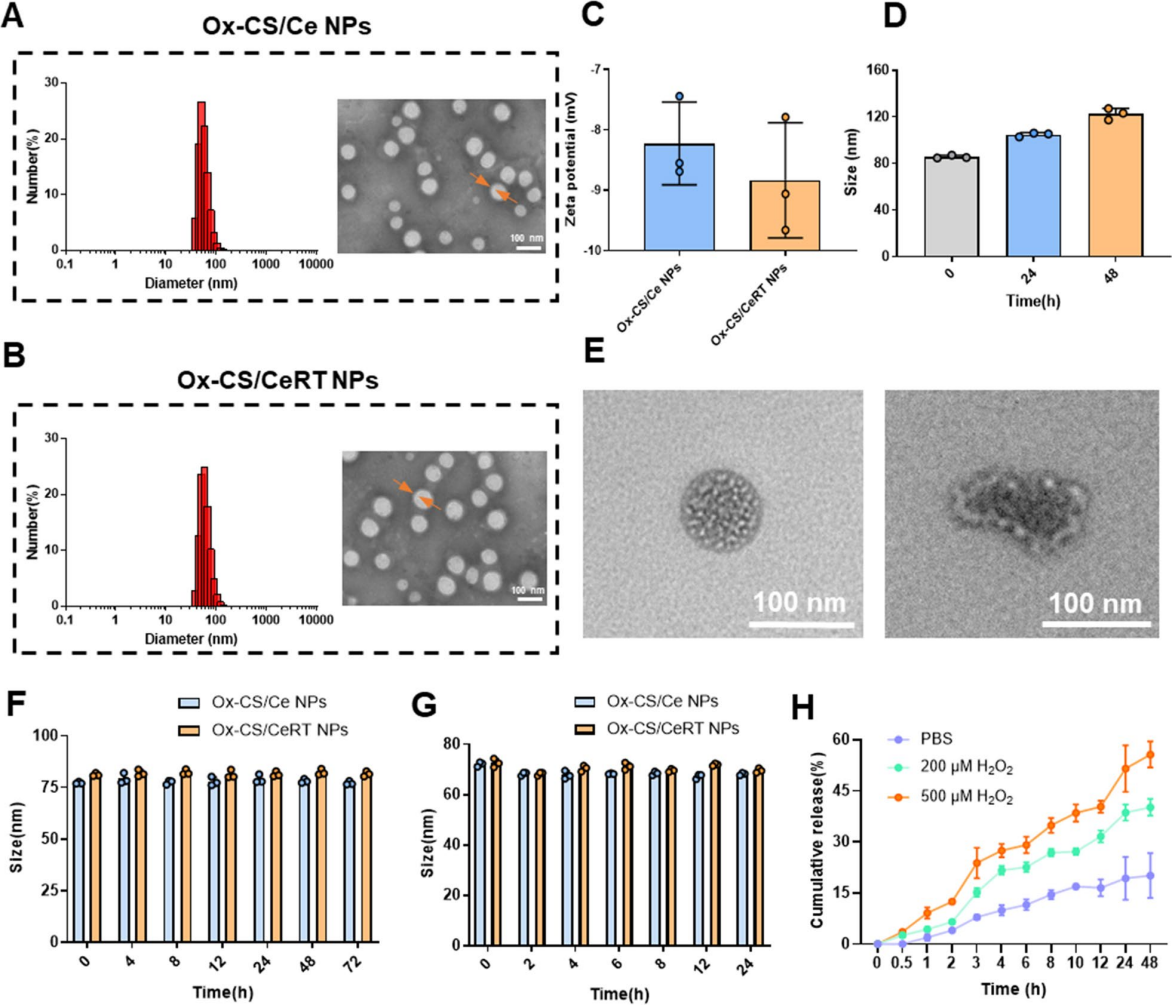
Characterization of Ox-CS/Ce NPs and Ox-CS/CeRT NPs. Size distributions and TEM images of **A** Ox-CS/Ce NPs and **B** Ox-CS/CeRT NPs, scale bar = 100 nm. Yellow arrows indicate core-shell structure. **C** Zeta potentials of Ox-CS/Ce NPs and Ox-CS/CeRT NPs. **D** Hydrodynamic particle size changes of Ox-CS/CeRT NPs under different times of incubation with H_2_O_2_. **E** Characteristic diagram of the morphological change of Ox-CS/CeRT NPs cultivated with 500 µM H_2_O_2_, scale bar = 100 nm. Stability of Ox-CS/CeRT NPs at **F** 4 °C (PBS) and **G** 37 °C (PBS containing 10% FBS). **H** Drug release kinetics of RT, incubated with 0, 200, and 500 µM H_2_O_2_ in PBS. Data are shown as mean ± SD (n = 3)

Ox-CS/Ce NPs and Ox-CS/CeRT NPs were prepared by thin-film dispersion method. Like other amphiphilic copolymers, Ox-CS could be self-assembly to form core-shell NPs. The presence of Ox-CS can make the drug stable, biocompatible, long half-life, and release drugs responsively. After successful coating with Ox-CS, the particle size of Ce NPs increased from 3.95 ± 0.53 nm to 87.9 ± 4.16 nm and the zeta potential changed from 19.5 ± 1.6 mV to − 8.2 ± 0.7 mV (Fig. 2C), possibly related to the positive charge of CS. After simultaneous encapsulation of Ce NPs and RT, the average particle size was 91.9 ± 0.43 nm and the zeta potential was − 8.84 ± 0.96 mV (Fig. 2C). The EE and DL of RT were 90.7% and 1.7%, respectively. Besides, The EE of Ce NPs in Ox-CS/Ce NPs and Ox-CS/CeRT NPs was 86.8% and 89.9%, while The DL of Ce NPs was 5.6% and 5.5%, respectively.

As shown in Additional file 4: Fig. S3, The FI-IR spectra of RT had a characteristic peak of the C=C stretching vibration at 1492 cm^−1^, C–N bending vibration at 1239 cm^−1^. The spectrum of Ox-CS/CeRT NPs (Additional file 4: Fig. S3C) exhibited typical peaks of Ox-CS at 1635 cm^−1^ (NH–CO stretching). However, the two RT peaks shifted to 1498 cm^−1^ and 1248 cm^−1^ which may have been due to the formation of hydrogen bond between Ox-CS and RT.

In addition, the TEM images revealed that both Ox-CS/Ce NPs and Ox-CS/CeRT NPs displayed a homogeneous spherical morphology. The core-shell structure also indicated that the material successfully modified the surface of the NPs (Fig. 2A, B). Moreover, high stability of Ox-CS/CeRT NPs was exhibited in PBS and serum (Fig. 2F, G).

It is well known that H_2_O_2_ oxidizes arylboronic esters [24], thus Ox-CS could degrade into pinacol borate, CS, and *p*-hydroxymethylphenol in the presence of H_2_O_2_ (overproduced at inflammatory tissues). Thus, the Ox-CS/CeRT NPs are supposed to be ROS-responsive. To determine the ROS-responsive ability of our copolymer, the diameter of Ox-CS/CeRT NPs incubation with 500 µM H_2_O_2_ was determined at a fixed time, the particle size increased time-dependently, indicating that the NPs were hydrolysed after H_2_O_2_ exposure (Fig. 2D). In addition, the TEM images showed that the regular globular morphology vanished following H_2_O_2_ incubation, also verifying the ROS responsiveness.

Besides, in vitro release of RT with different H_2_O_2_ concentrations was also investigated. Figure 2H shows the time-dependent release curve. As the H_2_O_2_ concentration increased, the release rate was considerably accelerated. These results indicated the excellent ROS responsiveness of these NPs. In summary, Ox-CS/CeRT NPs were identified as a novel ROS-sensitive system for the treatment of inflammatory disorders.

### In vitro cellular uptake of Ox-CS/CeRT NPs

As pulmonary vascular endothelial cells are not the only main target cells of injury, but also active inflammatory and effector cells, HUVECs were employed for the in vitro cell experiments.

Before biological evaluation, we examined the in vitro cellular toxicity of RT, Ox-CS, Ox-CS/Ce NPs and Ox-CS/CeRT NPs. Following a 24-h incubation at various doses, Ox-CS/Ce NPs did not exhibit cytotoxicity at concentrations of 2 µM. For RT, concentrations below 5 nM were non-toxic. Ox-CS/CeRT NPs have shown the same result (Additional file 5: Fig. S4). Therefore, 2 µM and 5 nM were selected for subsequent in vitro experiments, respectively. To establish an in vitro model of oxidative stress damage, HUVECs were treated with various concentrations of H_2_O_2_. Based on these results, 200 µM H_2_O_2_ was selected for treatment with HUVECs to serve as a control for the study of anti-oxidant activity in vitro (Additional file 5: Fig. S4C).

**Fig. 3:**
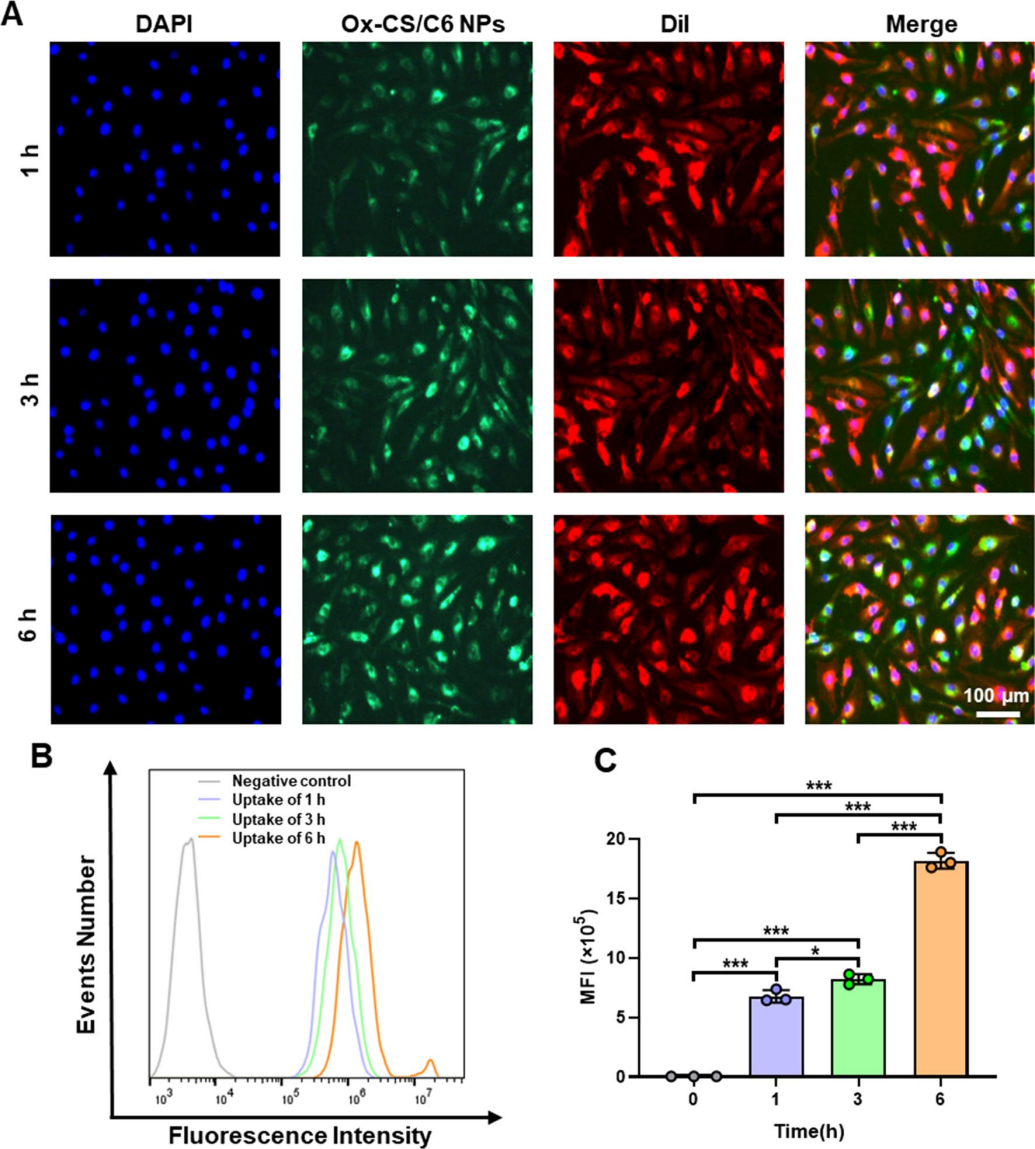
Cellular internalization against HUVECs. **A** Representative fluorescence microscopy image of HUVECs incubated with Ox-CS/C6 NPs (green) for different times (1, 3, 6 h). Nuclei were stained with DAPI (blue), while cell membrane were stained with DiI (red), scale bar = 100 μm. **B** Representative peak graph and **C** quantification of mean fluorescence intensity (MFI) in HUVECs after incubation with Ox-CS/C6 NPs for varied times performed by flow cytometry. n ≥ 3 per group. Data are shown as mean ± SD. *P < 0.05, **P < 0.01, ***P < 0.001

To analyse the uptake behavior of Ox-CS/CeRT NPs by HUVECs, fluorescence microscopy and flow cytometry were used. After treatment with Ox-CS/C6 NPs for different durations, significant green fluorescence was observed. With prolonged incubation, the fluorescence intensity gradually increased (Fig. 3A). Quantification by flow cytometry confirmed that NPs were internalised into cells in a time-dependent manner (Fig. 3B, C).

### Anti-oxidant and anti-inflammatory activities of Ox-CS/CeRT NPs in vitro

**Fig. 4:**
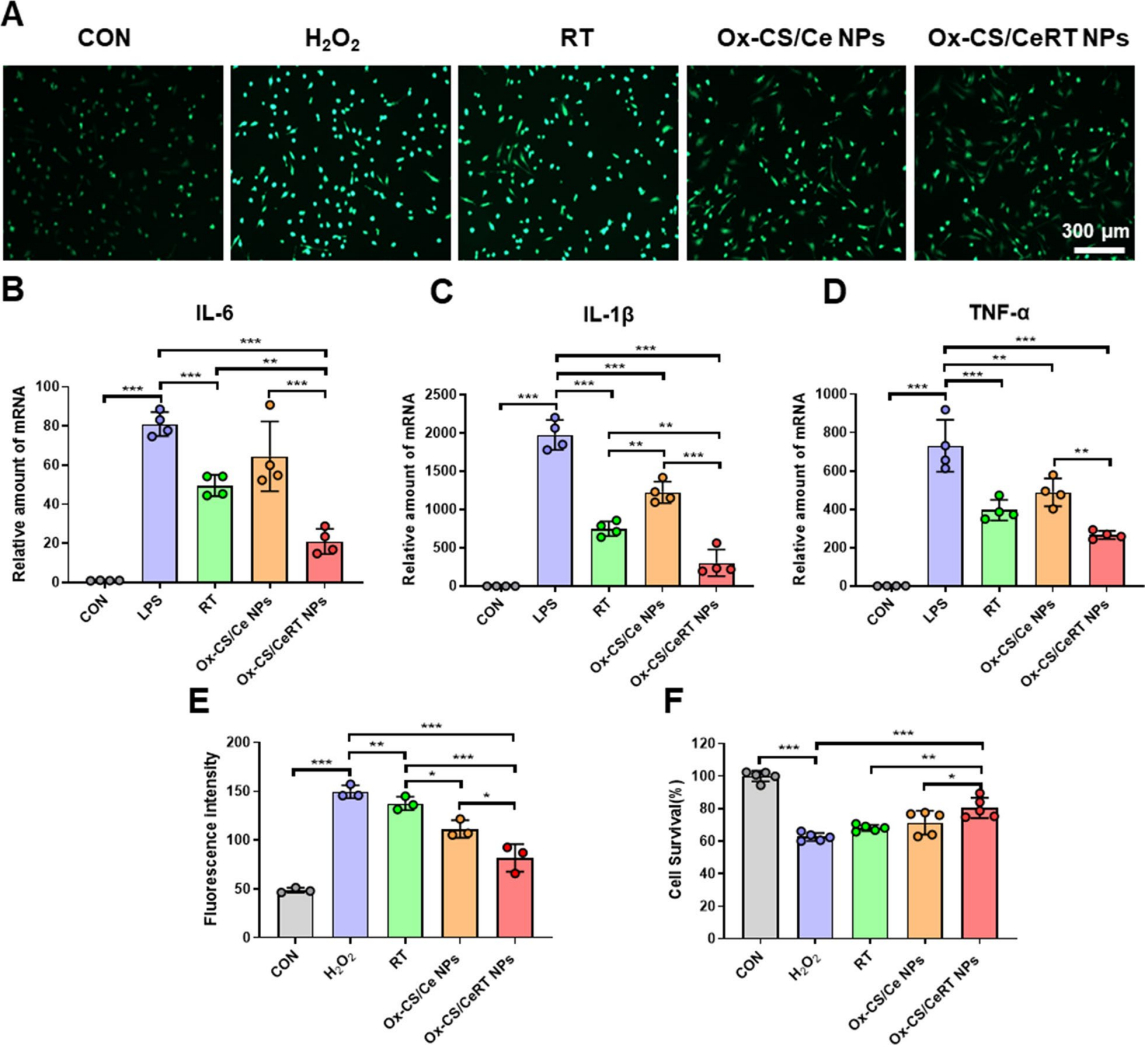
In vitro anti-oxidant and anti-inflammatory capacity of Ox-CS/CeRT NPs. **A** Intracellular ROS of H_2_O_2_-stimulated HUVECs after different interventions for 12 h was observed via fluorescent microscopy, scale bar = 300 μm. **B**–**D** The mRNA levels of pro-inflammatory cytokines in HUVECs after LPS treatment and different interventions for 4 h. Cytokine gene expression was normalized to GAPDH. **E** Semi-quantitative results of intracellular ROS. **F** Cell viability of H_2_O_2_-stimulated HUVECs after incubation with RT, Ox-CS/Ce NPs, Ox-CS/CeRT NPs for 24 h. n ≥ 3 per group. Data are shown as mean ± SD. *P < 0.05, **P < 0.01, ***P < 0.001

In the early stages of ALI, the overproduction of ROS causes oxidative stress in the tissue. Sustained oxidative stress can induce cellular and tissue damage [25], trigger inflammatory storms and amplify oxidative stress [26]. Therefore, we investigated whether Ox-CS/CeRT NPs can inhibit the ROS generation in HUVECs. Intracellular ROS was determined by fluorescence microscopy with DCFH-DA as the ROS probe. The level of intracellular ROS dramatically increased, as indicated in Fig. 4A. (***p < 0.001) after H_2_O_2_ stimulation as a probe by DCFH-DA that emits green fluorescence. In contrast, fluorescent signals of DCFH-DA were significantly attenuated after treatment of Ox-CS/Ce NPs or Ox-CS/CeRT NPs, particularly in the case of Ox-CS/CeRT NPs-treated cells (***p < 0.001). The Semi-quantitative results indicated that treatment with Ox-CS/CeRT NPs could successfully suppress the generation of intracellular ROS.

It is widely acknowledged that substantial levels of pro-inflammatory cytokines are required for the initiation and progression of the inflammatory cascade [27]. RT-qPCR was used to examine the expression of downstream pro-inflammatory cytokines at the gene level in LPS-stimulated HUVECs. As shown in Fig. 4B–D, LPS stimulation leads to a markedly increase in IL-6, IL-1β, and TNF-α (***p < 0.001). After treatment with RT, Ox-CS/Ce NPs, or Ox-CS/CeRT NPs, the mRNA expression levels were remarkably downregulated. Apparently, the Ox-CS/CeRT NPs group displays the best inhibition ability due to the combined effect of RT and Ce NPs among these groups.

### Anti-apoptotic activity of Ox-CS/CeRT NPs in vitro

High H_2_O_2_ concentrations would significantly increase cellular oxidative stress and excessive inflammatory responses, ultimately leading to apoptosis [28]. Accordingly, the anti-apoptotic capacity of Ox-CS/CeRT NPs was studied in vitro. The effect of Ox-CS/CeRT NPs on the viability of H_2_O_2_-induced HUVECs was investigated using the CCK-8 method. As shown in Fig. 4F, an obvious cytotoxicity was detected after exposure to H_2_O_2_ for 24 h. When treated with Ox-CS/CeRT NPs, the cell viability was significantly increased (80.5 ± 6.3% compared to free RT and Ox-CS/Ce NPs (***p < 0.001).

**Fig. 5:**
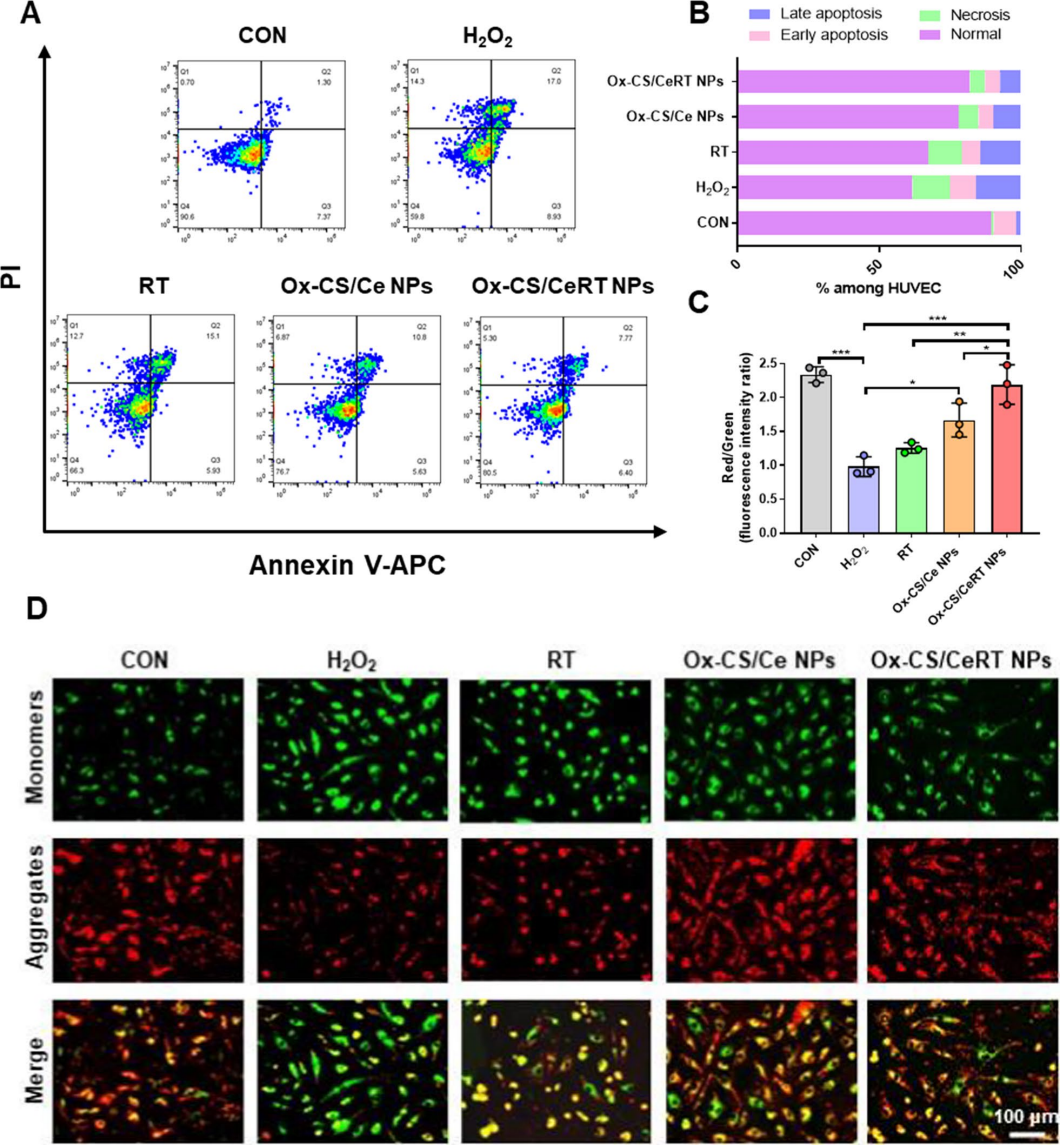
In vitro anti-apoptotic capacity of Ox-CS/CeRT NPs. **A** Typical flow cytometry profiles and **B** quantitative analysis of fluorescence intensity in HUVECs. **C** Semi-quantitative results of mitochondrial membrane potential (Δψm) changes of mitochondria. **D** Representative fluorescence microscopy image of Δψm changes in H_2_O_2_-stimulated HUVECs after different interventions for 24 h, scale bar = 100 μm. n = 3 per group. Data are shown as mean ± SD. *P < 0.05, **P < 0.01, ***P < 0.001

To further explore the anti-apoptotic effect of Ox-CS/CeRT NPs, flow cytometric analysis was performed using an Annexin V-APC/PI detection kit. Early apoptotic cells were those labelled with Annexin V-APC (Annexin V-APC+/PI−), whereas late apoptotic cells were those stained with both dyes (Annexin V-APC+/PI+). Owing to injury with 200 µM H_2_O_2_ for 24 h, the apoptotic rate was as high as nearly 40%; however, this rate could be markedly reduced to 20% via Ox-CS/CeRT NPs treatment, indicating a preventive effect in H_2_O_2_-induced apoptosis in vitro (Fig. 5A, B).

A decrease in membrane potential is seen as an early indicator of apoptosis since mitochondria are involved in the process. JC-1 probe was used for the determination of Δψ m. The decrease in Δψ m can be easily detected based on the shift in fluorescence from red to green. This shift can be regarded as an indicator for detecting early-stage apoptosis [29]. A lower ratio of JC-1 red/green indicates a higher degree of apoptosis. Compared to the control, the red/green fluorescence ratio was reduced to nearly 30% after H_2_O_2_ stimulation. However, this reduction was reversed by treatment with Ox-CS/CeRT NPs and was significantly higher than the groups subjected to RT and Ox-CS/Ce NPs-treatment (***p < 0.001) (Fig. 5C, D), suggesting that Ox-CS/CeRT NPs had the best ability to maintain mitochondrial function and thus anti-apoptosis.

Collectively, these data demonstrated that Ox-CS/CeRT NPs can efficiently inhibit pro-inflammatory cytokine expression and oxidative stress, indicating a significant promise for ALI therapy with these biomaterials.

### In vivo biodistribution of Ox-CS/CeRT NPs

**Fig. 6:**
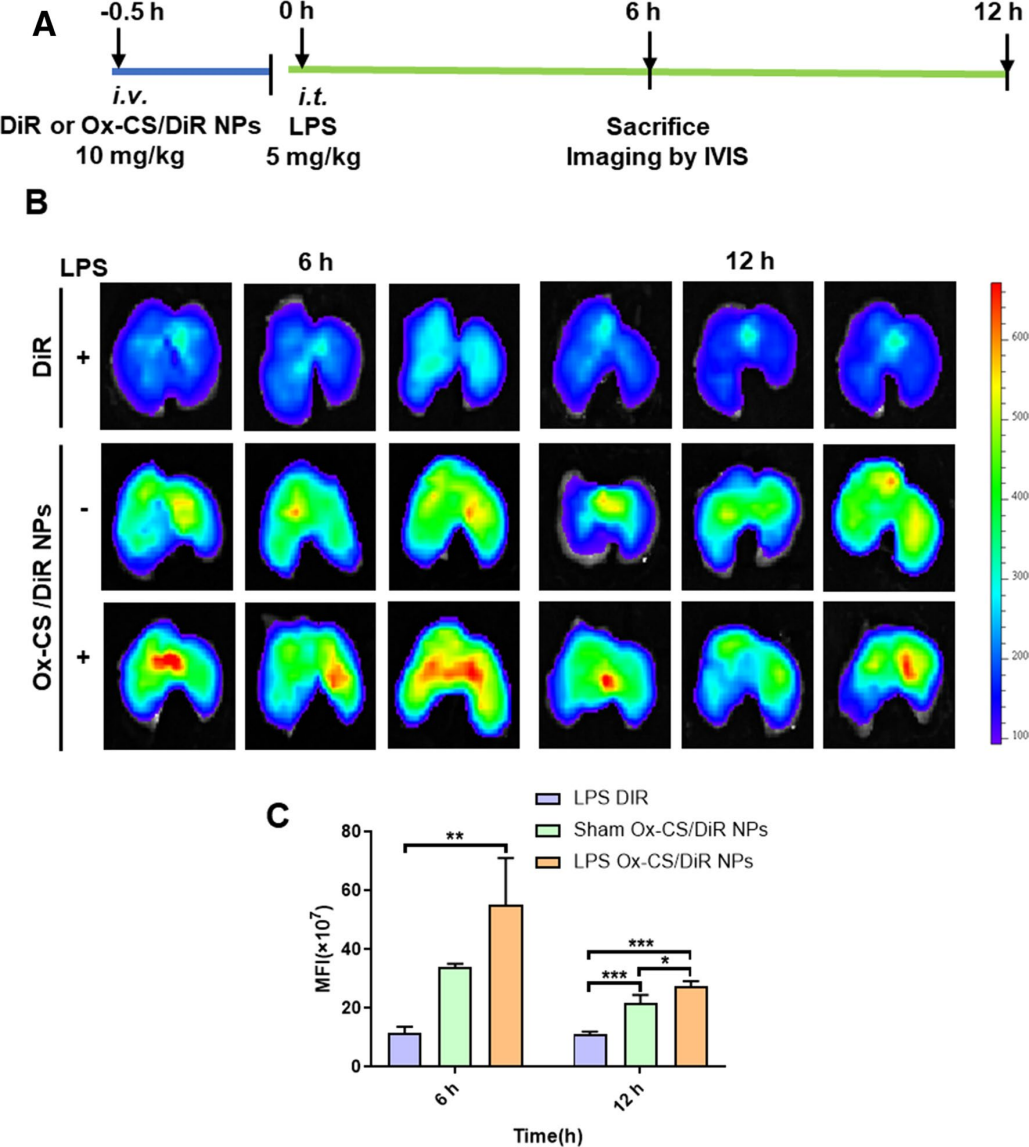
In vivo bio-distribution. **A** Experimental protocol for evaluating the targeting of NPs to inflammatory lungs. **B** The lung fluorescence intensity in free DiR or Ox-CS/DiR NPs treated ALI mice and Ox-CS/DiR NPs treated sham-operated mice at different times. **C** The semi-quantitation of fluorescence intensity in **B**. n = 3 per group. Data are shown as mean ± SD. *P < 0.05, **P < 0.01, ***P < 0.001

DiR and DiR-loaded NPs (Ox-CS/DiR NPs) were intravenous administered to ALI mice and the distribution of Ox-CS/CeRT NPs was examined. Figure 6A shows the schedule for the animal experiments.

After intravenous administration of free DiR or Ox-CS/DiR NPs for 6 and 12 h, the lung tissues were collected for imaging using IVIS. As shown in Fig. 6B, C, due to the EPR effect in inflamed tissues, the Ox-CS/DiR NPs treated group displayed significantly stronger fluorescence than free DiR in the ALI mouse (**p < 0.01). Despite a tendency of decreasing fluorescence, we continued to monitor the Ox-CS/DiR NPs treated group’s fluorescence for a long period of time (12 h). This fluorescence decrease may be related to fluorescence quenching and dye degradation. The quantitative analysis of IVIS demonstrated that the accumulation of DiR in the lung for mice treated with Ox-CS/DiR NPs was fivefold higher than that for the free DiR group. These results demonstrated that Ox-CS functionalization could enhance Ox-CS/DiR NPs accumulation in lung tissue in vivo.

In addition, the distribution of the fluorescent signals in the liver, kidney, and lung at 6- and 12-h post-injection was also shown in Additional file 6: Fig. S5. In comparison to the free DiR group, the Ox-CS/DiR NPs group’s liver and kidney fluorescence signals were noticeably weaker. These findings confirm that NPs can decrease drug accumulation in other organs, which may reduce toxicity and side effects.

### In vivo therapy of ALI

**Fig. 7:**
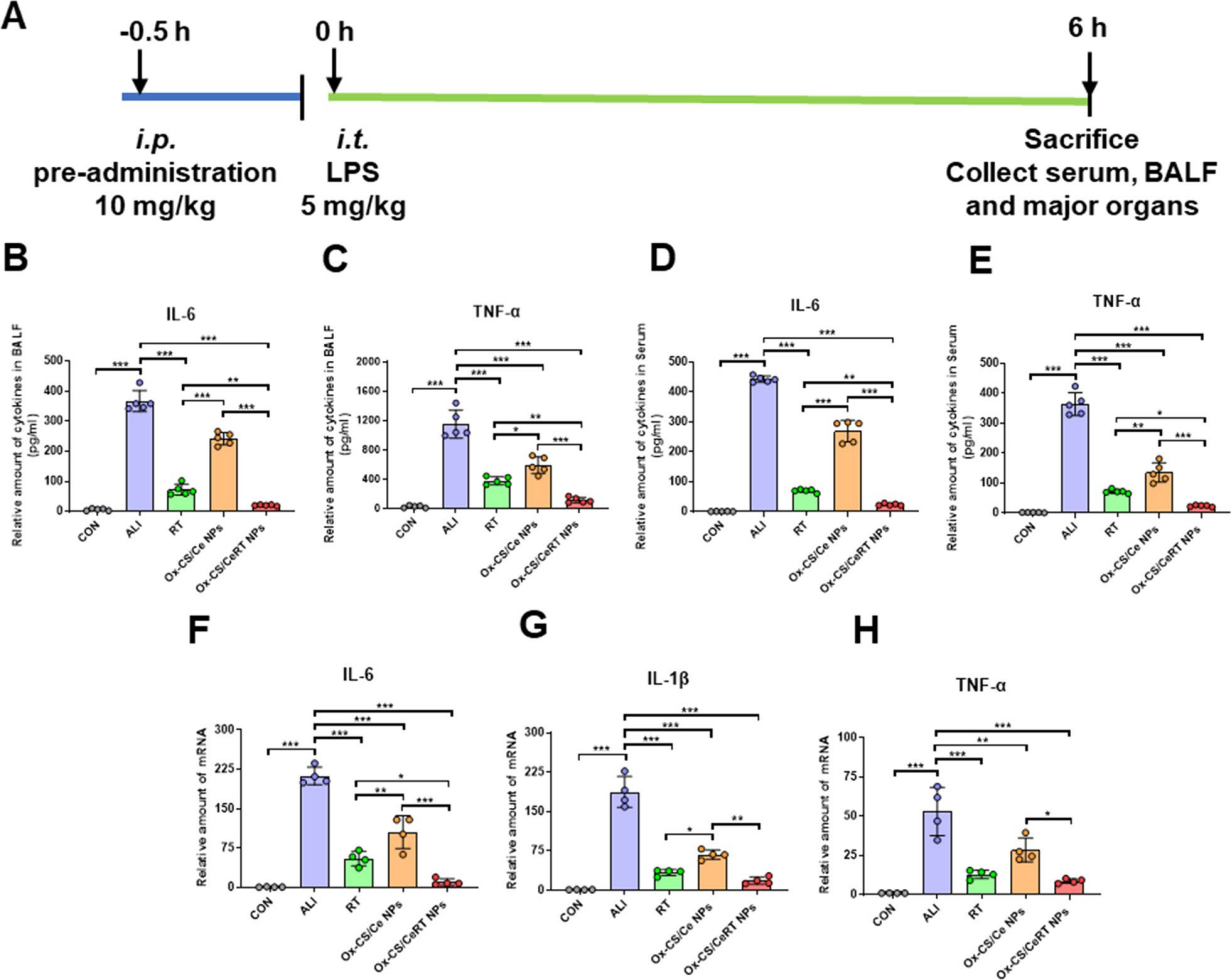
In vivo anti-inflammatory assessments of Ox-CS/CeRT NPs. **A** Treatment schedule. Levels of pro-inflammatory factors in BALF (**B** IL-6 and **C** TNF-α) or serum (**D** IL-6 and **E** TNF-α). The transcription levels of **F** IL-6, **G** TNF-α and **H** IL-1β in the lung tissues. Data were normalized to those of GAPDH. n ≥ 4 per group. Data are shown as mean ± SD. *P < 0.05, **P < 0.01, ***P < 0.001

Finally, we evaluate the therapeutic efficacy of Ox-CS/CeRT NPs in an LPS-induced ALI mouse model, which is one of the typical diseases characterized by an excessive inflammatory cytokine storm and ROS generation. Figure 7A shows the experimental scheme.

A significant characteristic of ALI is the disruption of pro-inflammatory mediators, which increases the inflammatory cascade response and causes severe injury [30]. BALF and circulating pro-inflammatory factors are reported to be elevated in mice after LPS stimulation, while pro-inflammatory gene expression is altered. Meanwhile, there is a significant infiltration of inflammatory cells in the lung tissue [31, 32].

Therefore, the levels of IL-6 and TNF-α in BALF and serum were measured through ELISA. Unsurprisingly, as shown in Fig. 7B–E, both inflammatory cytokines were noticeably increased in ALI mice (over 300-fold higher than in normal mice; ***P < 0.001). Similar to the in vitro assay, pro-inflammatory cytokine levels were reduced in all treatment groups., with the most significant decline observed in the Ox-CS/CeRT NPs treatment group (***P < 0.001). Furthermore, the mRNA levels of pro-inflammatory cytokines in the lung tissues were also assessed using RT-qPCR. Similarly, Ox-CS/CeRT NPs treatment leads to the most significant mRNA downregulation of all pro-inflammatory cytokines due to the ingenious combination of RT and Ce NPs (***P < 0.001) (Fig. 7F–H).

**Fig. 8:**
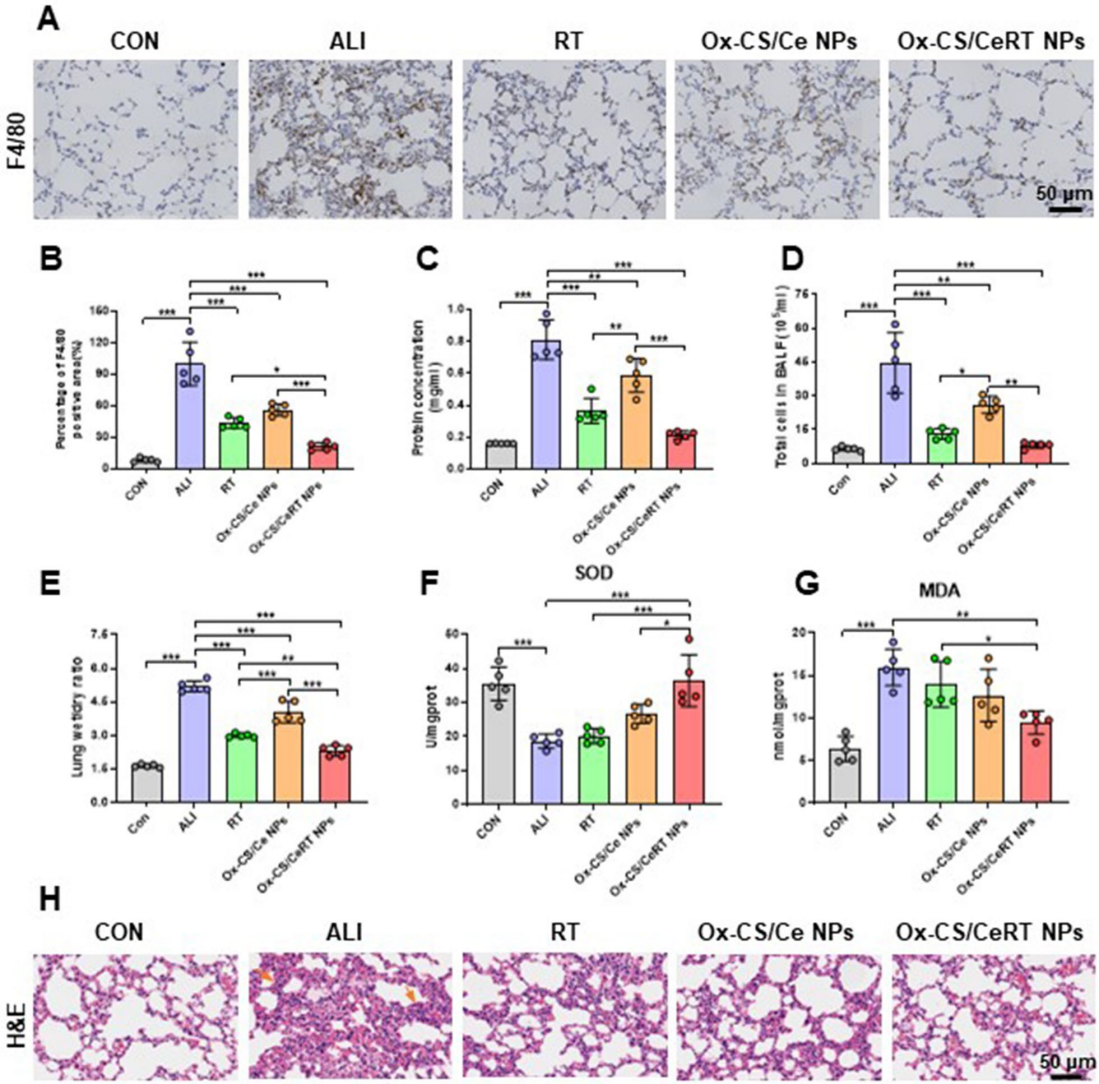
Ox-CS/CeRT NPs inhibited oxidative stress and morphological changes in lung tissue. **A** Immunohistochemical staining of the macrophage marker F4/80 (brown) in mouse lung tissue. Scale bar = 50 μm. **B** Quantification of F4/80 positivity. **C** Protein concentration in BALF. **D** Total cells in BALF. **E** Lung wet/dry ratio. **F** SOD activity and **G** MDA level in the lung tissue. **H** H&E-stained pathological sections of lung tissue. Scale bar = 50 μm. n = 5 per group. Data are shown as mean ± SD. *P < 0.05, **P < 0.01, ***P < 0.001

Immunohistochemical staining was performed using a macrophage-specific F4/80 antibody to assess macrophage infiltration. In the lung interstitial regions of ALI mice, F4/80 positive macrophages dramatically increased (***P < 0.001). However, this increase was significantly attenuated by all treatment groups, especially in the Ox-CS/CeRT NPs treatment group (***P < 0.001). According to these findings, Ox-CS/CeRT NPs have excellent anti-inflammatory properties (Fig. 8A, B).

Alveolar capillary barrier permeability is increased by LPS stimulation which results in an increased amount of total cells in the BALF [33]. Consequently, the cell count in the BALF was considered one of the important parameters to assess the effectiveness of ALI therapy. Therefore, we determined the protein concentration (Fig. 8C) and total cell count (Fig. 8D), all treatment groups exhibited a decrease in the protein concentration and cell count (**P < 0. 01), and the Ox-CS/CeRT NPs group has the lowest index, compared to the saline group (***P < 0. 001). One typical characteristic of ALI is pulmonary edema, which is featured by the elevated wet/dry weight ratio. Compared to the single drug group, the wet/dry weight ratio of the Ox-CS/CeRT NPs group completely recovered, which shows the most effective therapeutic impact (***P < 0. 001) (Fig. 8E).

SOD is a crucial enzyme that scavenges superoxide anion radicals and protects cells from damage [34]. MDA is used to evaluate lipid peroxidation, which reflects the damage degree in the body indirectly. We evaluated the levels of SOD and MDA to examine how Ox-CS/CeRT NPs affected oxidative stress in vivo. As shown in Fig. 8F, SOD levels were markedly reduced in the ALI group. All treatments improved the SOD levels, and Ox-CS/CeRT NPs displayed the best therapeutic efficacy (***p < 0.001). The MDA content in the ALI group increased. Additionally, a notable decrease was observed in the Ox-CS/CeRT NPs group (**p < 0.01) (Fig. 8G). These two findings demonstrate the potent anti-oxidant properties of Ox-CS/CeRT NPs. In summary, the Ox-CS/CeRT NPs play a critical role in inhibiting pro-inflammatory cytokines and oxidative stress for efficient treatment of lung injury.

Furthermore, the combined anti-inflammatory and anti-oxidant therapeutic efficacy of Ox-CS/CeRT NPs was assessed using H&E staining of lung tissues. According to the findings of lung tissue H&E staining, serious structural disruption and significant thickening of the alveolar walls were revealed for LPS-induced mice. Conversely, all therapy groups result in the repair of lung tissue, where abnormal pathological morphology has been alleviated to some degree (Fig. 8F). Surprisingly, the Ox-CS/CeRT NPs treatment group shows a substantially superior effect compared to single therapy.

### Evaluation of biosafety

As shown in Additional file 7: Fig. S6A, H&E staining of major mouse organs (heart, liver, spleen and kidney) clearly display normal histological morphologies. Liver and kidney function indicators represented by albuminous aminotransferase (ALT), aspartate aminotransferase (AST) and blood urea nitrogen (BUN) showed to be normal (Additional file 7: Fig. S6B–D).

Additionally, even when the concentration of Ox-CS/CeRT NPs was as high as 0.4 mM, the haemolysis rate of rat red blood cells was still below 5% (Additional file 7: Fig. S6E), confirming that Ox-CS/CeRT NPs has good blood compatibility. Consequently, Ox-CS/CeRT NPs shown high safety to blood and organ tissues.

## Conclusion

In conclusion, we developed a novel ROS-responsive nano-delivery system composed of Ox-CS to encapsulate RT and Ce NPs for ALI therapy. In particular, this obtained Ox-CS/CeRT NPs was able to release drug activated by high ROS level existing in inflammatory tissues. In vitro studies have shown that Ox-CS/CeRT NPs can efficiently scavenge ROS and prevent the generation of downstream pro-inflammatory cytokines in inflammatory endothelial cells. Consistently, the treatment of Ox-CS/CeRT NPs in vivo ALI model is significantly more effective for improving the diseased pulmonary tissue structure than single drug of RT or Ce NPs, through synergistically exerted anti-inflammatory and anti-oxidant effects. Therefore, the Ox-CS/CeRT NPs could be a promising candidate for clinical ALI therapy.

### Supplementary Information

**Additional file 1: Table S1.** qPCR primer pairs used in this study.

**Additional file 2: Figure S1.** Synthesis scheme of Ox-CS.

**Additional file 3: Figure S2.** ^1^H-NMR spectra of **A** CS, **B** CDI-PBAP, and **C** Ox-CS.

**Additional file 4: Figure S3.** The FI-IR spectra of **A** RT, **B** Ox-CS/Ce NPs and **C** Ox-CS/CeRT NPs.

**Additional file 5: Figure S4.** Effect of different interventions in a series of concentrations of **A** Ox-CS/Ce NPs, **B** RT, **C** Ox-CS, **D** Ox-CS/CeRT NPs, and **E** H_2_O_2_ on cell viability.

**Additional file 6: Figure S5.** **A** Representative fluorescence images and **B** semi-quantitation of fluorescence intensity of main organs collected by each group of mice at 6-h and 12-h postinjection.

**Additional file 7: Figure S6.** Biosafety assessment. **A** H&E-stained pathological sections of major organs from mice. Scale bar = 50 μm. Concentrations of **B** ALT, **C** AST, and **D** BUN in the ALI mice after the treatment of Ox-CS/Ce NPs.

## Data Availability

All data generated or analysed during the study period are included in the paper and/or Additional files. Additional data relevant to this study are available upon request.
