# Supplementary figures and images for: Synergistic anti-oxidant and anti-inflammatory effects of ceria/resatorvid co-decorated nanoparticles for acute lung injury therapy
Source: J Nanobiotechnology. 2023 Dec 21;21:502. doi: 10.1186/s12951-023-02237-y (PMC10740228; doi:10.1186/s12951-023-02237-y)

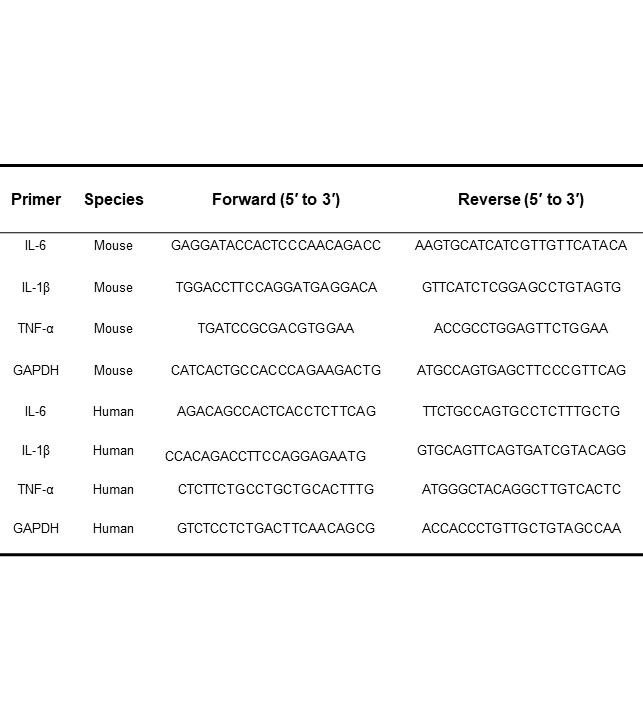

Supplement: Supplementary file 1 — Additional file 1: Table S1. qPCR primer pairs used in this study. [file 12951_2023_2237_MOESM1_ESM.jpg]

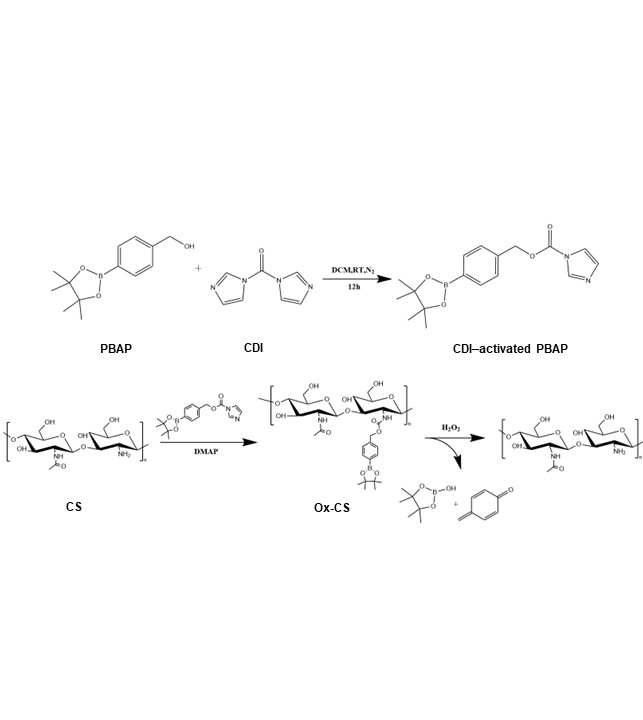

Supplement: Supplementary file 2 — Additional file 2: Figure S1. Synthesis scheme of Ox-CS. [file 12951_2023_2237_MOESM2_ESM.jpg]

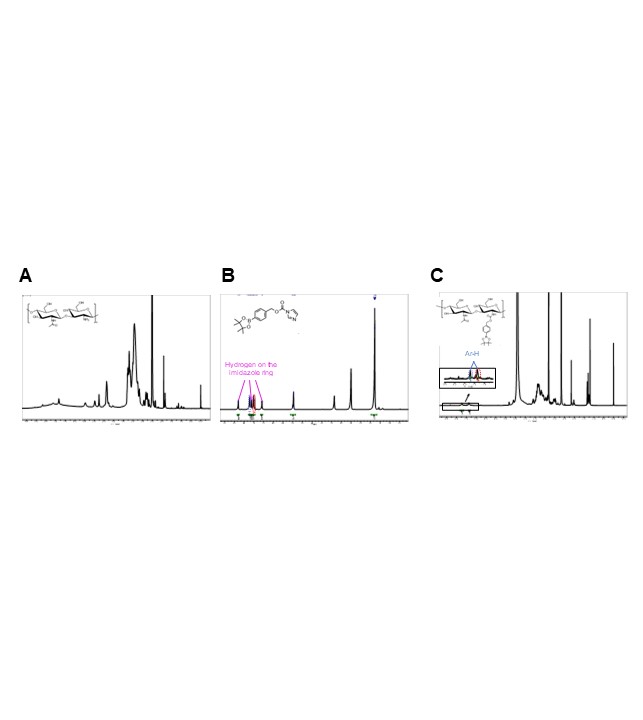

Supplement: Supplementary file 3 — Additional file 3: Figure S2. 1H-NMR spectra of A CS, B CDI-PBAP, and C Ox-CS. [file 12951_2023_2237_MOESM3_ESM.jpg]

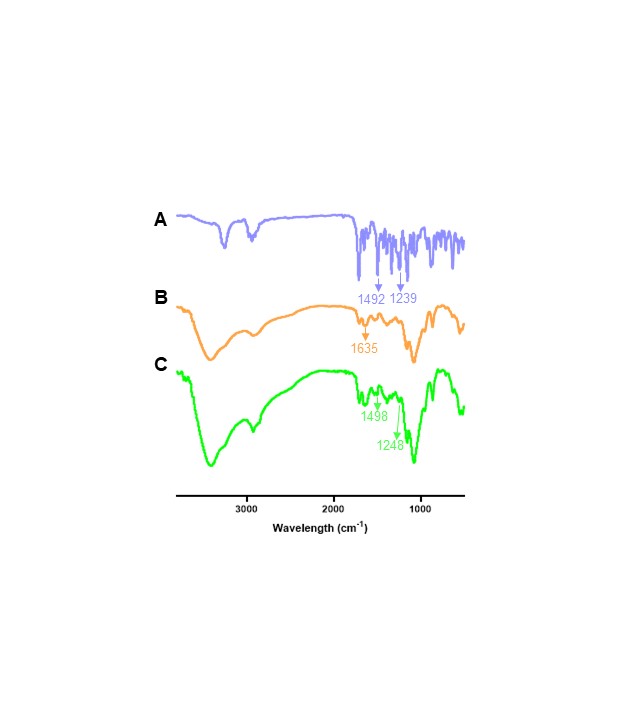

Supplement: Supplementary file 4 — Additional file 4: Figure S3. The FI-IR spectra of A RT, B Ox-CS/Ce NPs and C Ox-CS/CeRT NPs. [file 12951_2023_2237_MOESM4_ESM.jpg]

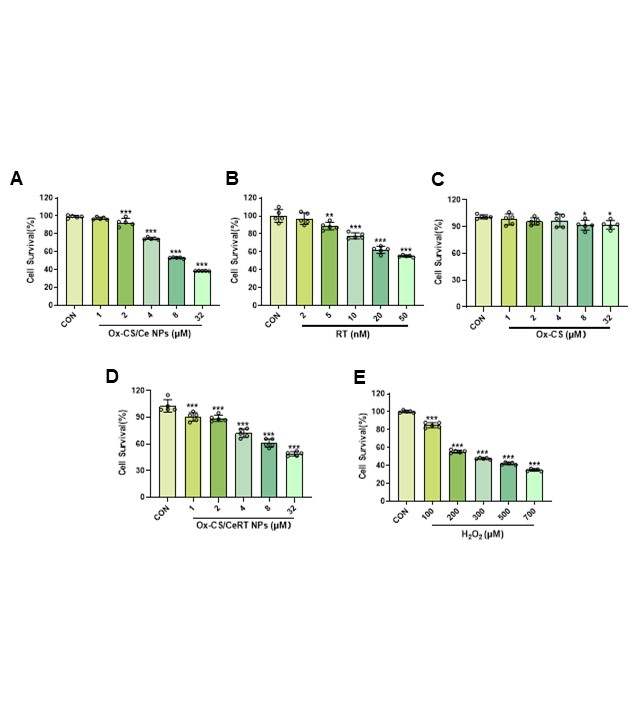

Supplement: Supplementary file 5 — Additional file 5: Figure S4. Effect of different interventions in a series of concentrations of A Ox-CS/Ce NPs, B RT, C Ox-CS, D Ox-CS/CeRT NPs, and E H2O2 on cell viability. [file 12951_2023_2237_MOESM5_ESM.jpg]

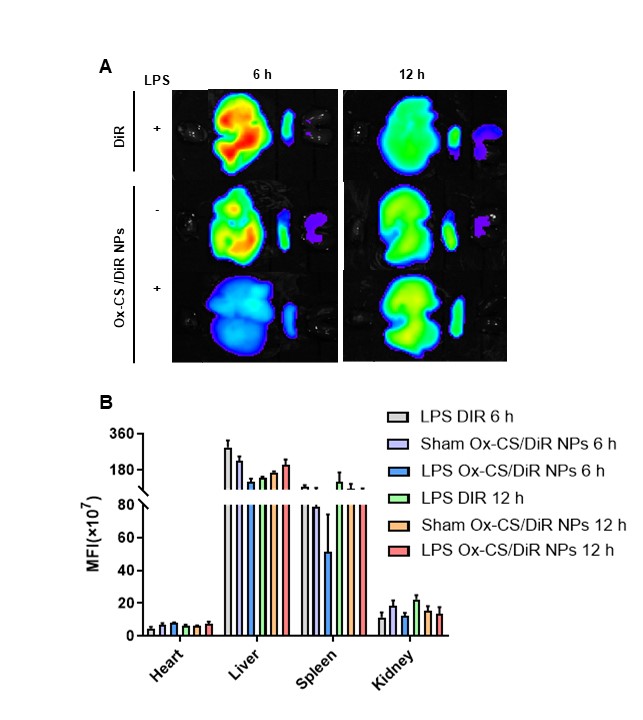

Supplement: Supplementary file 6 — Additional file 6: Figure S5. A Representative fluorescence images and B semi-quantitation of fluorescence intensity of main organs collected by each group of mice at 6-h and 12-h postinjection. [file 12951_2023_2237_MOESM6_ESM.jpg]

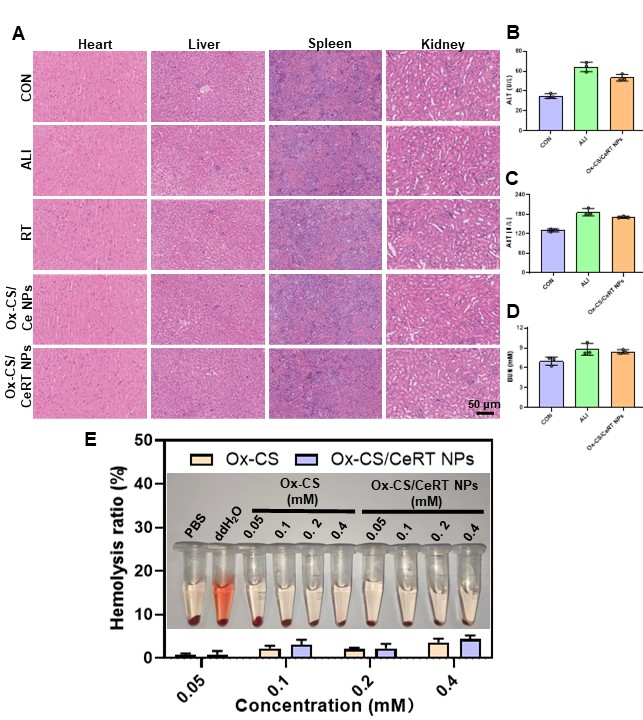

Supplement: Supplementary file 7 — Additional file 7: Figure S6. Biosafety assessment. A H&E-stained pathological sections of major organs from mice. Scale bar = 50 μm. Concentrations of B ALT, C AST, and D BUN in the ALI mice after the treatment of Ox-CS/Ce NPs. [file 12951_2023_2237_MOESM7_ESM.jpg]
